# Demonstrative Midwifery

**Published:** 1850-09

**Authors:** 


*Demonstrative Midwifery.*—We received, just as we were making up the Editorial matter for this Number, a letter from a distinguished member of the medical profession, a portion of which we cannot resist the temptation of inserting, relating, as it does, to a subject with which, by this time, our readers are somewhat familiar. The letter was not written with a view to publication, and we have only a presumption of the author's willingness that we should make any editorial use of it. Under these circumstances we feel obliged to withhold the name of our highly respected correspondent.

The anxieties and trials of the first case of Midwifery, so graphically and humorously portrayed, in the following extract, are sufficiently common, and hundreds of the most eminent of the Profession could make (if they would) confessions of a similar experience. The following is the portion of the letter referred to:—

"August 26th, 1850.

"I do hope and trust that this method of teaching obstetrics, now fairly begun, will not be abandoned. You have no doubt heard frequent confessions of the embarrassment and fright endured by young men in the conduct of their first case, and perhaps might add one more to the number from your own experience. I shall never forget mine. I was left alone with a poor Irish woman and one crony, to deliver her child. The "*dolores concussentes*" were in full vigor, and I thought it necessary to call up before me every circumstance I had learned from books, as at all possible in this critical moment. I must examine, and I did—but whether it was head or breech, hand or foot, man or monkey, that was defended from my uninstructed finger, by the distended membranes, I was as uncomfortably ignorant, with all my learning, as the fœtus itself that was making all this fuss. To add to my confusion, there were the poor patient and her friend "doctoring" me every minute, and striving to extort an opinion as to when the woman "would get to bed." But, in spite of my learned awkwardness, she did "get to bed," and the child was born. Fortunately, it was a natural labor. I had no difficulty in tying the cord and removing the infant. And then to hear the blessings showered upon my skill, and the prayers for the mother that bore me! It would have done your seventeen friends good. I assure you, there was no exposure in this case, for if the poor woman had been as nude as a model artist, I could have seen nothing then.

"However, as I said, the child was happily born, and I was overpowered with compliments, lavished with all the volubility of two Milesians. The nurse ran for a half pint, that "the docthur might dhrink the baby," while my doctorship sat in state, grinning from ear to ear, that I had escaped the commission of murder, and in danger of exploding with conceit at my wonderful achievement. But my triumph was of short duration. The nurse began to hint it was time for the after-birth to come away; and I, careful to ascertain that the mystical fifteen minutes had elapsed, made an attempt to bring it, but it would not come. Here was a fix—hemorrhage, hour-glass contraction, inverted uterus, and what not, if I used too much force, and retained placenta with its train of consequences, if I should fail in bringing it away. I pulled all I dared, and the woman became more significantly urgent every moment, until I was in danger of being sacrificed to the cause of "modest" science, when, happily for all parties, my preceptor dropped in, and with five words of *clinical demonstrative* instruction, every difficulty was removed, and I was enabled to complete the delivery. I took some pains to inquire of my fellow students during the remainder of my term of pupilage, if they, in their first case, had succeeded in bringing away the placenta without assistance—the answer was invariably, no, unless the uterine contractions had been sufficient to expel it from the os externum without manual aid.

"Now, if in those days we had had the benefit of Dr. White's *clinique*, what a deal of mental suffering would have been saved to me, and what a serious risk to the poor woman—nay, more—one woman, without the slightest risk to herself, would have been sufficient for the initiation of twenty students; whereas, under the old system it required twenty for the same purpose, and with twenty times the risk.

"If my opinion with regard to this matter, is of any consequence to you, I would, by all means, urge you to persevere. If more things could be physically demonstrated in the various departments of our profession, how incalculably would the science be advanced, and humanity benefitted! Show every thing you can to your pupils. The more numerous the "*oculis fidelibus subjecta*," the fewer will be the "*demissa per aures*." So far from abandoning the system of demonstrative midwifery, I would exhibit *every* stage of labor as well as the last, as far as is possible. It strikes me a speculum might be contrived that would exhibit every step of parturition, from the first perceptible dilatation of the os tincae to the final expulsion of the fœtus. I make this suggestion for the benefit of Dr. White, who is, of course, much more competent to decide upon its practicability than myself. Very truly, yours, &c.,

—— ———."

"Prof. A. Flint, Buffalo, N. Y."

